# Neuroimaging findings of inborn errors of metabolism: urea cycle disorders, aminoacidopathies, and organic acidopathies

**DOI:** 10.1007/s11604-023-01396-0

**Published:** 2023-02-02

**Authors:** Mikako Enokizono, Noriko Aida, Akira Yagishita, Yasuhiro Nakata, Reiko Ideguchi, Ryo Kurokawa, Tatsuo Kono, Toshio Moritani, Harushi Mori

**Affiliations:** 1 Department of Radiology, Tokyo Metropolitan Children’s Medical Center, 2-8-29 Musashidai, Fuchu, Tokyo 183-8561 Japan; 2 Department of Radiology, Kanagawa Children’s Medical Center, Yokohama, Kanagawa Japan; 3 Department of Neuroradiology, Tokyo Metropolitan Neurological Hospital, Fuchu, Tokyo Japan; 4 Department of Radioisotope Medicine, Atomic Bomb Disease Institute, Nagasaki University, Nagasaki, Japan; 5 The Division of Neuroradiology, Department of Radiology, University of Michigan, Ann Arbor, MI USA; 6 Department of Radiology, School of Medicine, Jichi Medical University, Shimotsuke, Tochigi Japan

**Keywords:** Inborn errors of metabolism, Urea cycle disorders, Aminoacidopathy, Organic acidopathy, Magnetic resonance spectroscopy

## Abstract

Although there are many types of inborn errors of metabolism (IEMs) affecting the central nervous system, also referred to as neurometabolic disorders, individual cases are rare, and their diagnosis is often challenging. However, early diagnosis is mandatory to initiate therapy and prevent permanent long-term neurological impairment or death. The clinical course of IEMs is very diverse, with some diseases progressing to acute encephalopathy following infection or fasting while others lead to subacute or slowly progressive encephalopathy. The diagnosis of IEMs relies on biochemical and genetic tests, but neuroimaging studies also provide important clues to the correct diagnosis and enable the conditions to be distinguished from other, more common causes of encephalopathy, such as hypoxia–ischemia. Proton magnetic resonance spectroscopy (^1^H-MRS) is a powerful, non-invasive method of assessing neurological abnormalities at the microscopic level and can measure in vivo brain metabolites. The present review discusses neuroimaging findings, including those of ^1^H-MRS, of IEMs focusing on intoxication disorders such as urea cycle disorders, aminoacidopathies, and organic acidopathies, which can result in acute life-threatening metabolic decompensation or crisis.

## Introduction and overview

Inborn errors of metabolism (IEMs) are genetic disorders caused by mutations in genes coding for proteins that function in metabolism. They present complicated, medical conditions often involving several organ systems. IEMs predominantly involving the central nervous system (CNS) are known as neurometabolic disorders. Most IEMs are inherited in an autosomal recessive manner and are rarely autosomal dominant or X-linked. While the types of IEMs vary widely, they are relatively common, having an overall incidence of about 1:2,500 [1]. Nonetheless, individual cases are rare, and their diagnosis is often challenging. However, early diagnosis of neurometabolic disorders is mandatory to initiate therapy and prevent permanent long-term neurological impairment or death.

IEMs can be classified into four major groups depending on the mechanism producing the clinical presentation: (1) intoxication disorders, (2) energy production disorders, (3) disorders of the biosynthesis and metabolism of complex molecules, and (4) neurotransmitter defects [2, 3]. Intoxication disorders are characterized by variable, symptom-free intervals followed by acute and/or chronic CNS intoxication symptoms. Due to the presence of these metabolites in the placenta and the mother's metabolism, newborns with these conditions are often asymptomatic. Subsequent deterioration is caused by the postnatal accumulation of toxic metabolites. The most important examples of intoxication disorders are urea cycle disorders (UCDs), aminoacidopathies, and organic acidopathies. Mitochondrial disorders, the most prevalent abnormalities of energy production, typically present with multisystemic symptoms affecting high metabolic rate tissues, such as the brain, heart, and skeletal muscles. Lysosomal and peroxisomal disorders are examples of biosynthetic and metabolic abnormalities; their symptoms are slowly progressive, permanent, and unrelated to diet. Neurotransmitter disorders, such as pyridoxine and pyridoxal 5'-phosphate dependency, are recognized as the underlying cause of severe metabolic encephalopathy that typically manifests in neonates. They influence monoamine synthesis as well as glycine and GABA metabolism [3].

The clinical course of neurometabolic disorders is very diverse. Some diseases develop as acute encephalopathy following infection or fasting while others develop as subacute or slowly progressive encephalopathy. Earlier clinical onset occurs in children who have more profound enzymatic defects (neonatal, infantile) whereas those with milder biochemical phenotypes typically experience symptoms later (in adolescence or adulthood). Acute encephalopathy includes severe intoxication disorders and mitochondrial disorders presenting with acute decompensation at younger ages, as well as neurotransmitter diseases. Acute decompensation includes lactic acidosis, ketosis or ketoacidosis, hyperammonemia, and hypoglycemia or hyperglycemia. The neurological manifestations include hypotonia, seizures, metabolic stroke with rapid onset of extrapyramidal movement disorders, and coma. Subacute and chronic encephalopathy involves mild intoxication disorders, mitochondrial disorders, lysosomal disorders, and peroxisomal disorders that develop at an older age. Their neurological manifestations include developmental delay, seizures, pyramidal and extrapyramidal movement disorders, and neurocognitive and behavioral problems. The diagnosis of IEMs relies on biochemical and genetic tests, but neuroimaging studies also provide important clues for diagnosis and can help distinguish these conditions from other causes of encephalopathy.

Proton magnetic resonance spectroscopy (^1^H-MRS) is a powerful non-invasive method of assessing neurological abnormalities at the microscopic level and measuring in vivo brain metabolites [4]. Moreover, ^1^H-MRS findings have the potential to be a biomarker for monitoring therapeutic efficacy in metabolic diseases and enable metabolic changes to be detected both in normal-appearing and diseased brain tissue with nonspecific injury patterns.

In the present article, we review neuroimaging findings, including those of ^1^H-MRS, while focusing on intoxication disorders such as UCDs, aminoacidopathies, and organic acidopathies, which can result in acute life-threatening metabolic decompensation or crisis.

## Technique and basic method of ^1^H-MRS for pediatric neurometabolic disorders

Accurate interpretation of ^1^H-MRS data necessitates high-quality spectra, characterized by a sufficient signal-to-noise ratio (SNR) and narrow peak linewidths. Factors impacting SNR include magnetic field strength, uniformity of the magnetic field, voxel size, sequence parameters, such as echo time (TE) and repetition time (TR), and the number of excitations (NEX). A 3-T scanner is preferable to a 1.5-T scanner because of its superior SNR and increased spectral resolution. To mitigate local magnetic field distortions caused by factors, such as air, bone, calcification, and blood products, the volume of interest (VOI) should ideally be located entirely within brain tissue. In cases where the spectrum is lacking adequate SNR, increasing the NEX may improve the results.

Single-voxel ^1^H-MRS is commonly recommended for most disorders, particularly pediatric neurometabolic disorders, as it offers high SNR and the capacity to identify various metabolites [5, 6]. VOI localization methods include sequences, such as point-resolved spectroscopy (PRESS) and stimulated echo acquisition mode (STEAM), with PRESS being more widely used because its SNR is twice as high as that of STEAM [7]. VOI of 8 mL or less is preferred for in vivo brain ^1^H-MRS [7]. We recommend placing the voxel in pathological lesions for imaging, particularly in acute lesions that exhibit restricted diffusion and regions with no apparent abnormal signal, such as deep gray matter (DGM) and/or the centrum semiovale (CS).

Notable brain metabolites observed by ^1^H-MRS include: n-acetyl aspartate (NAA) and N-acetylaspartylglutamate (tNAA) at 2.0 ppm, glycerophosphocholine (including choline-containing compounds) and phosphocholine (collectively referred to as Cho) at 3.2 ppm, and creatine and phosphocreatine (collectively referred to as Cr) at 3.0 ppm, which may be quantified at TE up to 280 ms. On short TE (20–35 ms), myo-inositol (mIns) at 3.5 ppm, combined glutamate (Glu) and glutamine (Gln) (Glx) at about 2.1 to 2.6 ppm, and lipid and/or macromolecule peaks at 0.9 and 1.3 ppm may be quantified. The main ^1^H-MRS peaks and their significance are as follows: NAA, a neuronal metabolite and in vivo biomarker of the presence of viable neurons or the assessment of parenchymal damage; Cr, a marker of the energetic reserve; Cho, a marker of cellular proliferation from increased membrane turnover and/or inflammation; and mIns, a glial metabolite and marker of gliosis. Elevated lactate (Lac), as a doublet peak at 1.3 ppm, reflects anaerobic glycolysis and is a nonspecific biomarker of several pathologies. In short TE, lipid and/or macromolecule peaks overlap the Lac peak, but lipid and/or macromolecule peaks disappear in intermediate TE (135–144 ms) and long TE (272–288 ms) [5, 7].

Short TE is the first choice since it allows the detection of more metabolites and has greater SNR than longer TE (intermediate or long TE). If feasible, the incorporation of longer TE acquisition should be considered for the assessment of Lac and metabolites with relatively longer T2. To avoid signal loss resulting from insufficient longitudinal relaxation, the TR should be set to 2000 ms for 3-T and 1500 ms for 1.5-T or greater [6, 7].

MRI and ^1^H-MRS may be complementary techniques for evaluating neurometabolic disorders. ^1^H-MRS offers a non-invasive, cost-effective approach, which can be easily incorporated as an add-on to clinical MRI (in as little as 3–5 min) and has the potential to enhance diagnostic accuracy and inform treatment decisions. Nonetheless, the interpretation of MR spectra in the developing brain requires caution as spectra are known to change continuously during brain maturation. Many studies have shown that NAA and Cr signals increase in childhood while Cho and mIns signals decline rapidly within the first year of life and continue to decline gradually until approximately 20 years of age [8].

## Urea cycle disorders (UCDs)

**Fig. 1:**
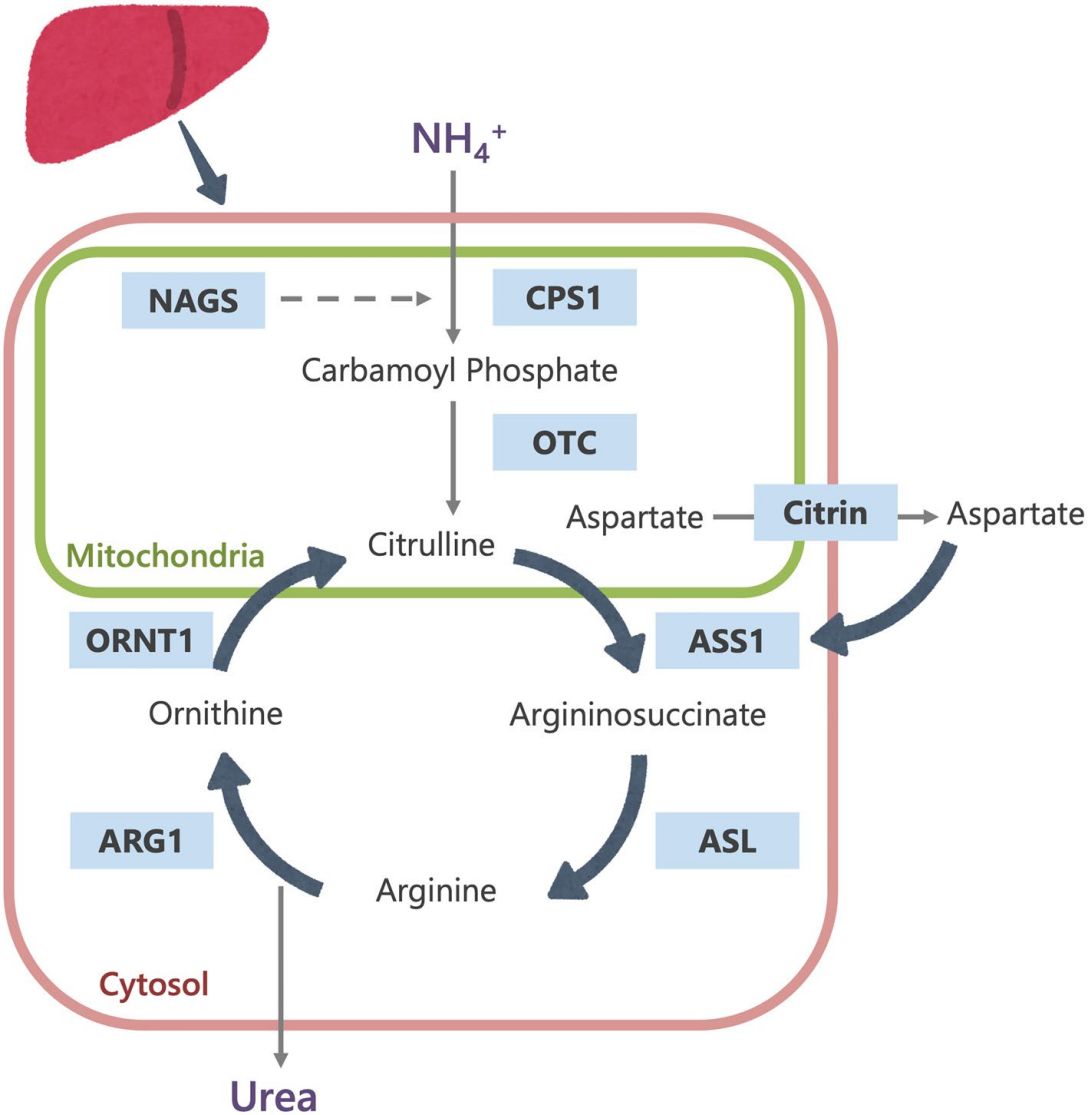
The urea cycle (*CPS1* Carbamoylphosphate synthetase I, *NAGS* N-acetyl glutamate synthetase, *OTC* ornithine transcarbamylase, *ASS1* argininosuccinic acid synthetase, *ASL* argininosuccinic acid lyase, ARG1 arginase, *ORNT1* ornithine transporter)

The urea cycle, which converts ammonia into urea, is a major pathway for the disposal of nitrogen in the body. It consists of (1) five catalytic enzymes (carbamoylphosphate synthetase I [CPS1], ornithine transcarbamylase [OTC], argininosuccinic acid synthetase [ASS1], argininosuccinic acid lyase [ASL], and arginase [ARG1]; (2) a cofactor-producing enzyme (N-acetyl glutamate synthetase [NAGS]); and (3) two amino acid transporters (ornithine translocase [ORNT1] and citrin) (Fig. 1) [9]. UCDs stem from inherited deficiencies of any one of the six enzymes or two transporters in the urea cycle pathway (CPS1, OTC, ASS1, ASL, ARG1, NAGS, ORNT1 or citrin). Table 1 summarizes the UCD types.

**Table 1:** Differentiation of urea cycle disorders

Disease	Gene/locus	Mode of inheritance	Enzyme defect	Clinical features
CPS1 deficiency	*CPS1* 2q34	AR	Carbamoyl phosphate synthetase I (CPS1)	Hyperammonemia
NAGS deficiency	*NAGS* 17q21.31	AR	N-acetyl glutamate synthetase (NAGS)	Hyperammonemia
OTC deficiency	*OTC* Xp11.4	XLR	Ornithine transcarbamylase (OTC)	Hyperammonemia
ASS1 deficiency (citrullinemia type I)	*ASS* 9q34.11	AR	Argininosuccinic acid synthetase (ASS)	Hyperammonemia
ASL deficiency (argininosuccinic aciduria)	*ASL* 7q11.21	AR	Argininosuccinic acid lyase (ASL)	Hyperammonemia, hepatomegaly, elevation of transaminases, trichorrhexis nodosa (fragile hair)
ARG1 deficiency (hyperargininemia)	*ARG1* 6q23.2	AR	Arginase (ARG)	Hyperammonemia, hyperargininemia, progressive spasticity
ORNT1 deficiency (HHH syndrome)	*SLC25A15* 13q14.11	AR	Ornithine transporter (ORNT1)	Hyperammonemia, chronic neurocognitive deficits, chronic liver dysfunction
Citrin deficiency (citrullinemia type II)	*SLC25A13* 7q21.3	AR	Citrin	NICCD in newborns, FTTDCD in older children, CTLN2 in adults

*HHH syndrome* hyperornithinemia–hyperammonemia–homocitrullinuria syndrome, *AR* autosomal recessive, *XLR* X-linked recessive, *NICCD* neonatal intrahepatic cholestasis caused by citrin deficiency, *FTTDCD* failure to thrive and dyslipidemia caused by citrin deficiency, *CTLN2* citrullinemia type II

## Clinical characteristics of UCDs

The location of the defective protein along the pathway affects the severity of urea cycle defects. As there is no effective secondary ammonia clearance system, severe deficiency or total absence of activity of any of the first four enzymes in the pathway (CPS1, OTC, ASS1 or ASL) or the cofactor producer (NAGS) results in the accumulation of ammonia and other precursor metabolites during the first few days of life. CPS1 deficiency is the most severe form of UCDs. NAGS deficiency symptoms are similar to CPS1 deficiency symptoms because NAGS inactivates CPS1. In X-linked dominant OTC deficiency, females are variably affected whereas males are always severely affected [9]. In milder or partial urea cycle enzyme deficiencies, ammonia accumulation can be triggered at nearly any time of life by illness or stress (e.g., surgery, prolonged fasting, peripartum period), resulting in multiple, mild increases in plasma ammonia concentration.

The initial symptoms of a neonate with hyperammonemia are nonspecific and may manifest as progressive irritability, lethargy, poor feeding, hypothermia or seizures. Individuals with partial enzyme deficiencies present symptoms later (in childhood, adolescence or adulthood) and have intermittent neurological dysfunction manifesting as movement disorders, seizures, ataxia, lethargy or confusion. In the mildest cases, the symptoms may be psychiatric, such as sleep disorder, delusions, hallucinations or psychosis.

No physical examination findings can be used to distinguish between the eight types of UCD; however, trichorrhexis nodosa, a beaded appearance of fragile hair, may suggest ASL deficiency [10], and progressive spasticity of the lower extremities may indicate ARG1 deficiency [11]. Some affected individuals with ASL deficiency have chronic hepatic enlargement and elevated transaminases. ARG1 deficiency causes hyperargininemia and may present with episodic hyperammonemia of varying degrees but is rarely severe enough to be life-threatening or to cause death. Untreated individuals with ARG1 deficiency experience a slowing of linear growth at age 1 to 3 years, followed by the development of spasticity, seizures, plateauing of cognitive development, and subsequent loss of developmental milestones [11].

ORNT1 and citrin deficiency may both cause hyperammonemia. The former, which is also known as hyperornithinemia-hyperammonemia-homocitrullinuria syndrome (HHH syndrome), may also involve chronic liver dysfunction. Citrin deficiency typically only manifests hyperammonemia in adolescents and adults with citrullinemia type II (CTLN2) but may also occur in infants with neonatal intrahepatic cholestasis caused by citrin deficiency (NICCD) and in older children with failure to thrive and dyslipidemia caused by citrin deficiency (FTTDCD) [12]. Beyond age 1 year, many children with citrin deficiency develop a protein-rich and/or lipid-rich food preference and an aversion to carbohydrate-rich foods. The symptoms of CTLN2 are often induced by alcohol or sugar intake, medication, and/or surgery.

Current newborn screening (NBS) panels in Japan using tandem mass spectrometry can detect abnormal concentrations of analytes associated with ASS1 deficiency and ASL deficiency. Citrin deficiency is screened for only in some local municipalities.

## Neuroimaging of UCDs

Hyperammonemia leads to CNS dysfunction. Ammonia diffuses freely across the blood–brain barrier and is quickly converted to glutamine, which is osmotically active, leading to astrocytic swelling and cytotoxic edema [13].

**Fig. 2:**
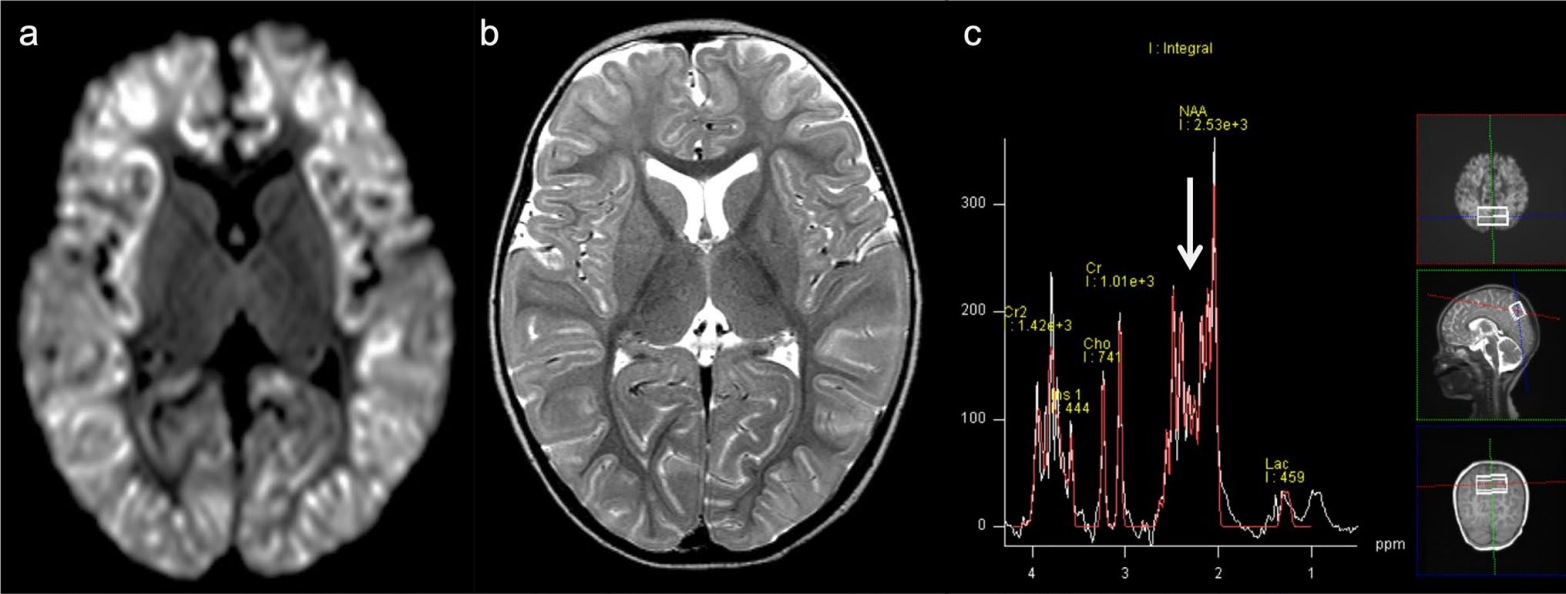
Ornithine transcarbamylase (OTC) deficiency. A 2-year-old, female patient with acute hyperammonemic encephalopathy. **a**, **b** DWI (**a**) and T2-weighted images (**b**) demonstrated a diffuse, scalloped ribbon pattern of injury to the cerebral cortices. **c** ^1^H-MRS (TE = 30 ms) of the affected bilateral parietal lobes indicated a slight elevation of Glx (arrow)

The neuroimaging pattern of UCDs in neonates is characterized by edema involving the cerebral cortex and the subcortical white matter and most severely affects the peri-rolandic, peri-insular cortex [3]. In addition, the basal ganglia may show edema (particularly the globi pallidi). Basal ganglia as well as the insular and peri-rolandic cortex are the most metabolically active regions in term neonates and consequently the most vulnerable areas [14]. The thalami are typically spared. The pattern of globi pallidi and putaminal involvement, while sparing the thalami, allows UCDs to be distinguished from hypoxic-ischemic encephalopathy (HIE). In contrast, in older children and adults, the insulae and cingulate gyri are most affected, and the peri-rolandic and occipital cortex are spared [15], mimicking herpes simplex virus encephalitis [16]. However, severe injury results in more diffuse, generalized edema with a nonspecific pattern. The so-called scalloped ribbon pattern of injury on DWI strongly supports the diagnosis of UCDs (Fig. 2) [3, 14]. Ultimately, severe, multicystic atrophy of the gray and white matter develops.

^1^H-MRS typically demonstrates an elevation of Lac, and the Gln-Glu complex (Glx) with short TE (Fig. 2) [2, 5, 17]. The magnitude of mIns, an osmotic buffer, correlates inversely with disease severity. Longstanding hyperammonemia decreases choline possibly by reducing the delivery of choline, which is synthesized in the liver [18]. These pathological changes may be reversed with treatment [4].

**Fig. 3:**
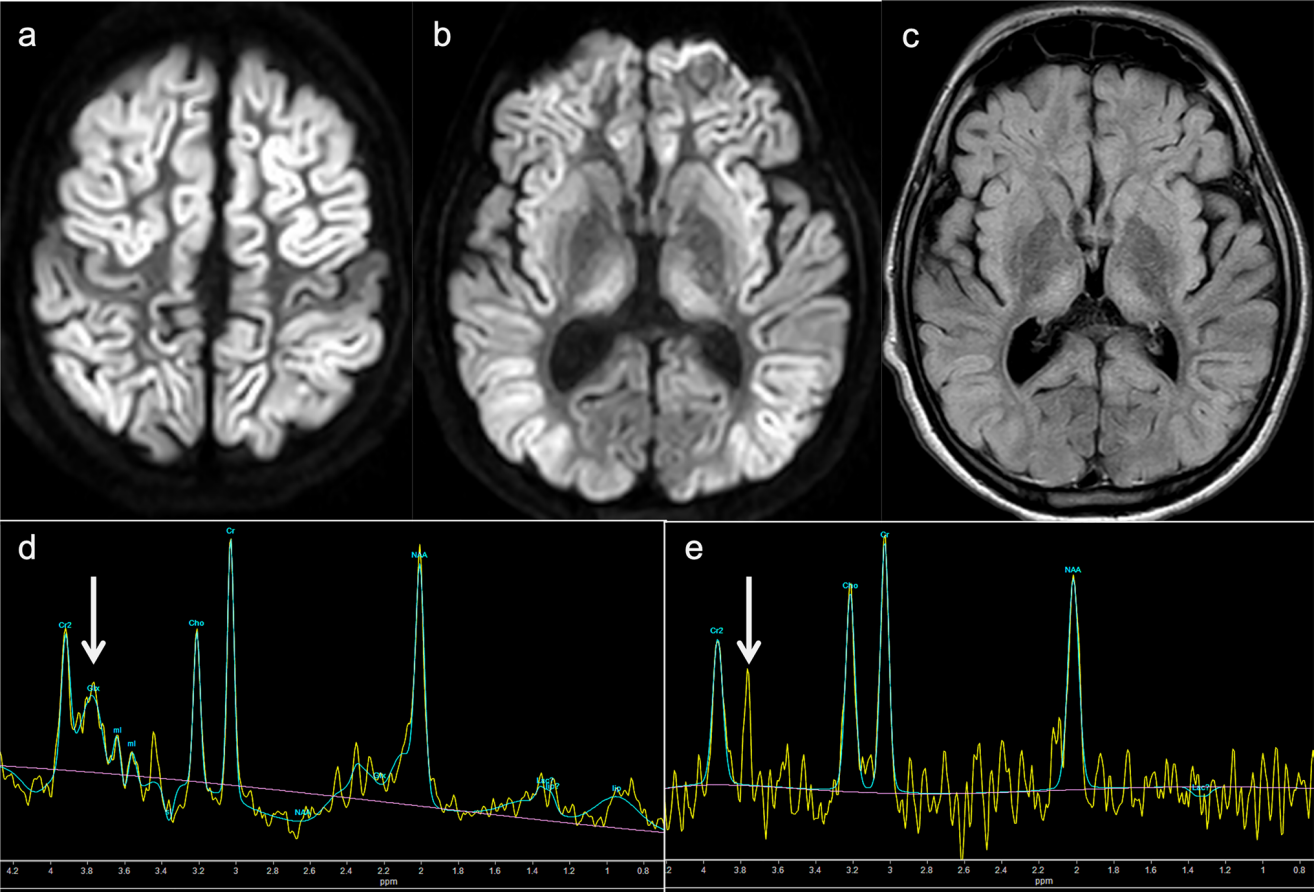
Arginase (ARG1) deficiency. A 16-year-old, male patient with developmental delay and epilepsy. Five days after receiving the COVID-19 vaccine, acute hyperammonemic encephalopathy developed. **a**-**c** DWI (**a**, **b**) and FLAIR images (**c**) demonstrated diffuse, high-signal areas in the bilateral cortices (the peri-central sulci are unaffected). Symmetrical, high-signal areas were also seen in the bilateral striata and medial thalami. Diffuse brain atrophy was also observed. **d**, **e** ^1^H-MRS (TE = 35, 144 ms) of the affected right thalamus displayed elevated arginine at 3.8 ppm, which was clearly visible at 144 ms (**e**) (arrows)

Distal UCD defects are less likely to present with acute hyperammonemic encephalopathy. Therefore, brain MRI may show normal or nonspecific atrophy. Gln does not increase, but other metabolites may; in ASL deficiency, guanidinoacetate may increase to 3.8 ppm [19], and in ARG1 deficiency the arginine level is around 3.87 ppm [20]. The arginine peak can be seen even in short TE, but in longer TE, the suppression of the Glx peak makes the peak more clearly visible (Fig. 3).

**Fig. 4:**
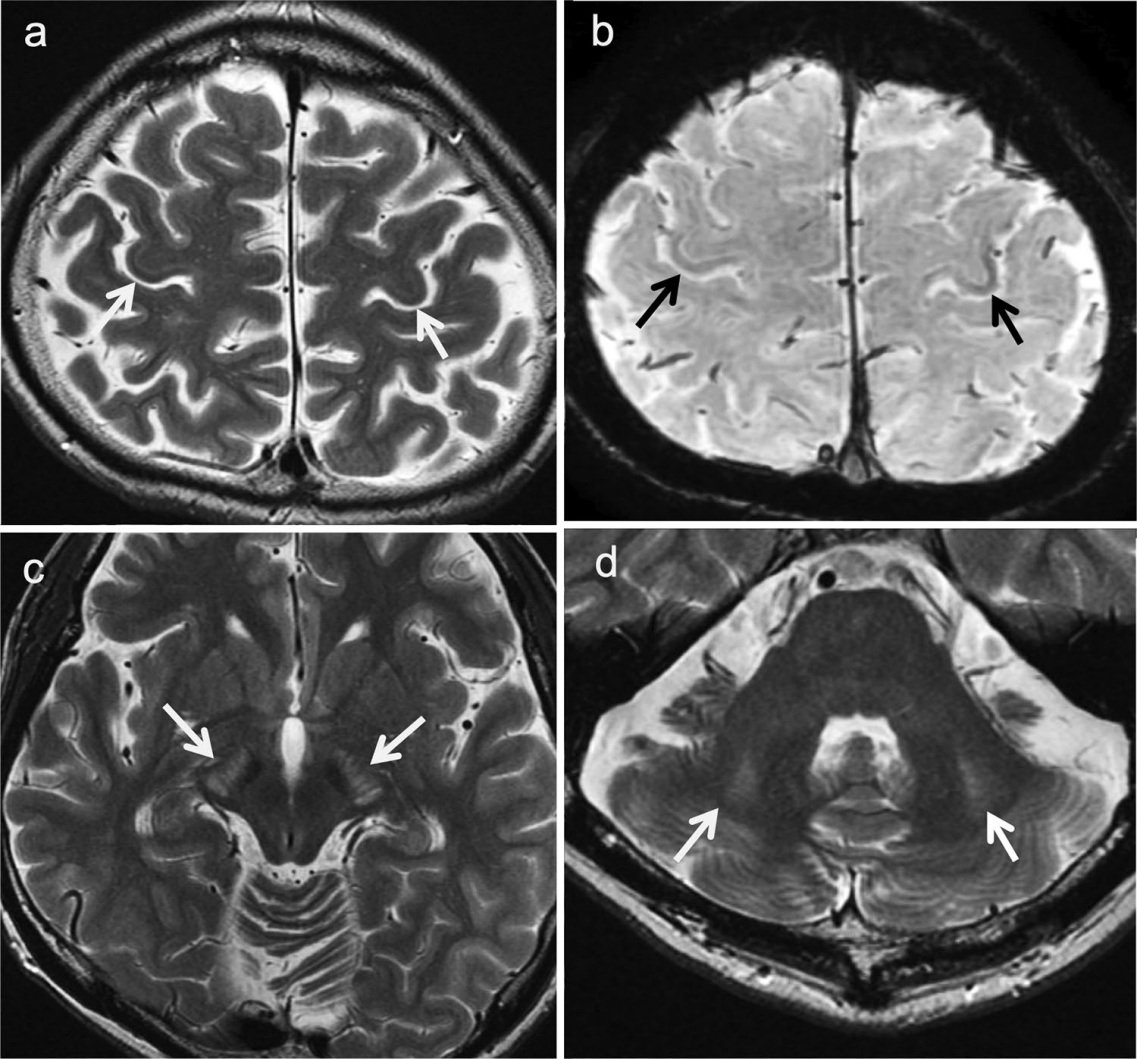
Citrullinemia type II (CTLN2) (chronic progressive type). A 42-year-old, male patient with epileptic seizures. The patient had hyperammonemia and hypercitrullinemia with normal liver function. **a**, **b** T2-weighted images (**a**) and SWAN (**b**) displayed bilateral, low signals in the deep, bilateral, precentral cortices (arrows). **c**, **d** T2-weighted images demonstrated hyperintensity in the bilateral cerebral peduncles and middle cerebellar peduncles (arrows). (Source: reference 21)

The neuroimaging findings of CTLN2 differ between the acute and the chronic progressive types of encephalopathy. In the latter, brain atrophy is visible on MRI. Symmetrical T2-weighted imaging shows areas of high signal intensity in the bilateral pyramidal tracts (precentral white matter, posterior limb of the internal capsules, and cerebral peduncles), middle cerebellar peduncles, and cerebellar white matter. In addition, areas of low signal intensity may be seen in the deep bilateral precentral cortices on T2-weighted imaging and SWAN (T2 Star Weighted MR ANgiography) (Fig. 4) [21]. The bilateral globi pallidi may also show areas of hyperintensity on T1-weighted imaging [22]. These findings are similar to those of chronic acquired hepatocerebral degeneration (hepatic encephalopathy) [23].

## Aminoacidopathies

### Maple syrup urine disease (MSUD)

Maple syrup urine disease (MSUD) is an autosomal recessive disorder of the catabolism of branched-chain amino acids (BCAA; leucine, isoleucine, and valine) caused by a deficiency of the branched-chain keto-acid (BCKA) dehydrogenase enzyme. Three causative genes (*BDKDHA* at 19q13.2, *BCKDHB* at 6q14.1, and *DBT* at 1p21.2) are known [24]. MSUD is classified into three subtypes: classic, intermediate, and intermittent. Classic MSUD, the most severe form, presents as asymptomatic in neonates but rapidly progressed to encephalopathy if left untreated. Early detection is vital as the initiation of therapy within the first five days of life is associated with better neurodevelopmental outcomes [25]. In contrast, children with the intermittent form of MSUD typically have normal growth and intellectual development during infancy and early childhood. However, they may experience metabolic crises triggered by infection or high protein intake, which may not be detectable by NBS [26].

**Fig. 5:**
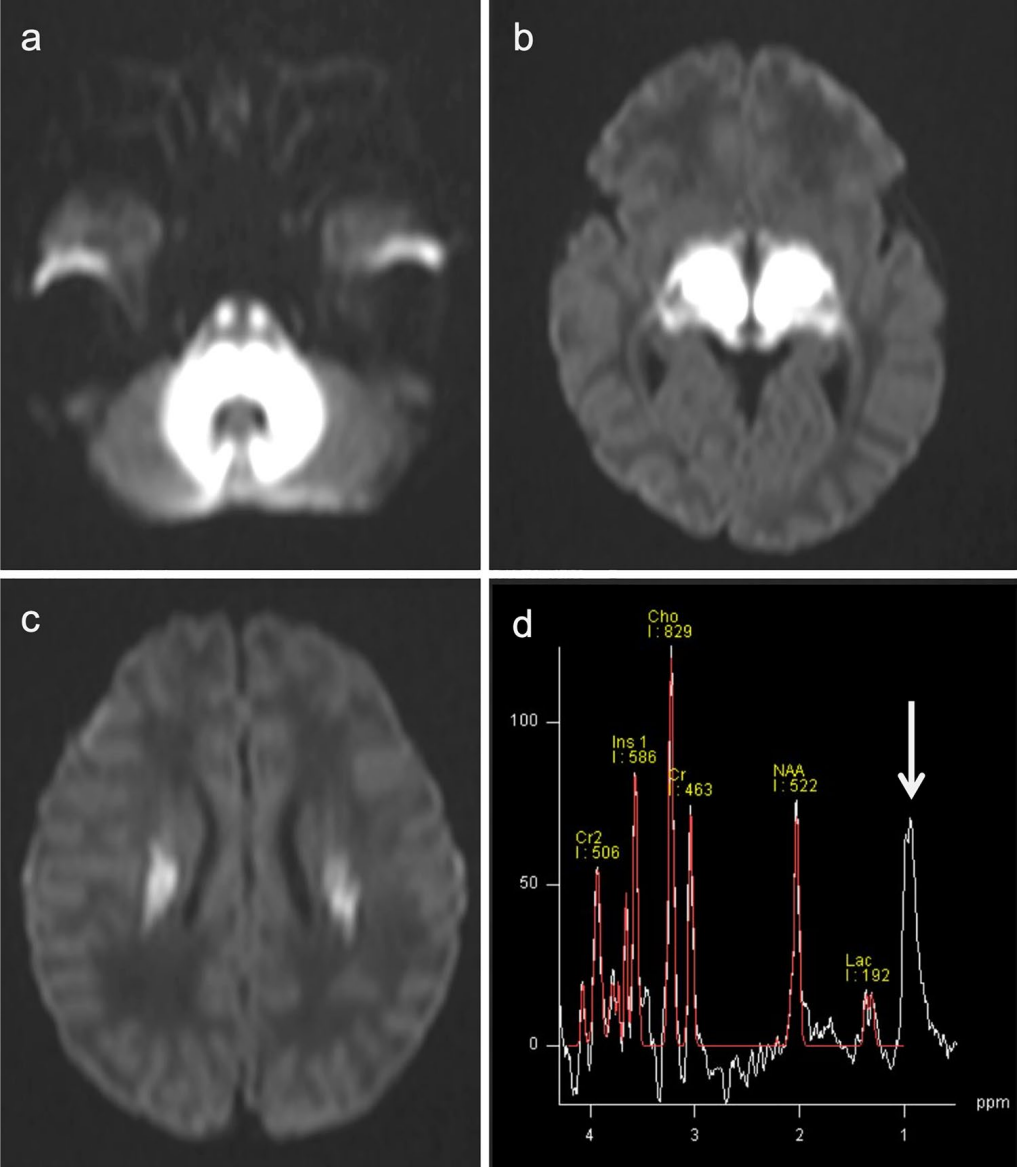
A male neonate with classic maple syrup urine disease (MSUD) during a metabolic crisis. **a**–**c** DWIs revealed marked diffusion restriction in the cerebellar white matter, dorsal pons, thalami, posterior limb of the internal capsules, globi pallidi, and central corona radiata. **d** ^1^H-MRS (TE = 30) of the left centrum semiovale demonstrated a broad, BCAA and BCKA peak at 0.9 ppm (arrow) (Source: reference 33)

The typical neuroimaging findings of classic MSUD are intramyelinic edema characterized by marked diffusion restriction along myelinated white matter (cerebellar white matter, dorsal brainstem, cerebral peduncles, posterior limb of the internal capsules, and peri-rolandic cerebral white matter), the thalami, and globi pallidi during metabolic crises in the neonatal period (Fig. 5). Electron microscopy studies in animal models of MSUD have revealed the formation of intramyelinic vacuoles resulting from the accumulation of water between the myelinic lamellae, which leads to intramyelinic edema [27]. Intramyelinic edema is thought to be caused by an energy failure resulting in a decreased Na^+^/K^+^ ATPase activity due to BCKA accumulation [3, 28]. The myelin sheath is composed of layers of myelin that form tight junctions with axons and isolate the periaxonal space and spaces between the myelin layers from other extracellular spaces. Intramyelinic edema, the non-neurotoxic edema that forms within these spaces, is characterized by restricted diffusion and reversibility of the condition without cell death. Intramyelinic edema alone is considered to be fully reversible whereas concurrent cellular edema results in an irreversible or partially reversible condition [29].

In addition to intramyelinic edema, vasogenic edema involving the unmyelinated brain structures may be observed. This second type of edema is caused by a dysfunction of the blood–brain barrier causing a global increase in water in the extracellular spaces and is present only during acute metabolic decompensation or a crisis [30, 31].

In the intermittent form of MSUD in late infancy and childhood, the intramyelinic edema is more extensive and resembles early Canavan disease because of the progression of myelination [32].

^1^H-MRS is diagnostic and displays a characteristic, broad peak complex at 0.9 ppm due to BCAA and BCKA elevation (Fig. 5) [33]. In addition to short TE, longer TE ^1^H-MRS is useful for eliminating the overlapping lipid peak at 0.9 ppm. A Lac peak (anaerobic glycolysis) and decreased NAA/Cr ratio may also be present.

## Nonketotic hyperglycinemia (NKH) or glycine encephalopathy

Nonketotic hyperglycinemia (NKH), also known as glycine encephalopathy, is an autosomal recessive disorder characterized by deficient glycine cleavage enzyme system (GCS) activity, which leads to the buildup of enormous amounts of glycine in various tissues of the body, including the brain. There are three known causative genes (*AMT* at 3p21.31, *GLDC* at 9p24.1, and *GCSH* at 16q23.2). Most patients with NKH exhibit symptoms in the neonatal period, including progressive lethargy, hypotonia, and myoclonic jerks, which rapidly progress to apnea, coma, and often death within the first two days of life [34]. Infants who survive generally have profound intellectual disabilities and intractable seizures. The neurotoxicity of glycine is thought to be related to its excitatory and inhibitory effects on glycine and N-methyl-D-aspartate receptors in the telencephalon and brainstem/spinal cord, respectively [35]. Furthermore, the GCS plays a vital role in neurogenesis [35, 36] and disruption of its activity can have detrimental effects on the neonate.

**Fig. 6:**
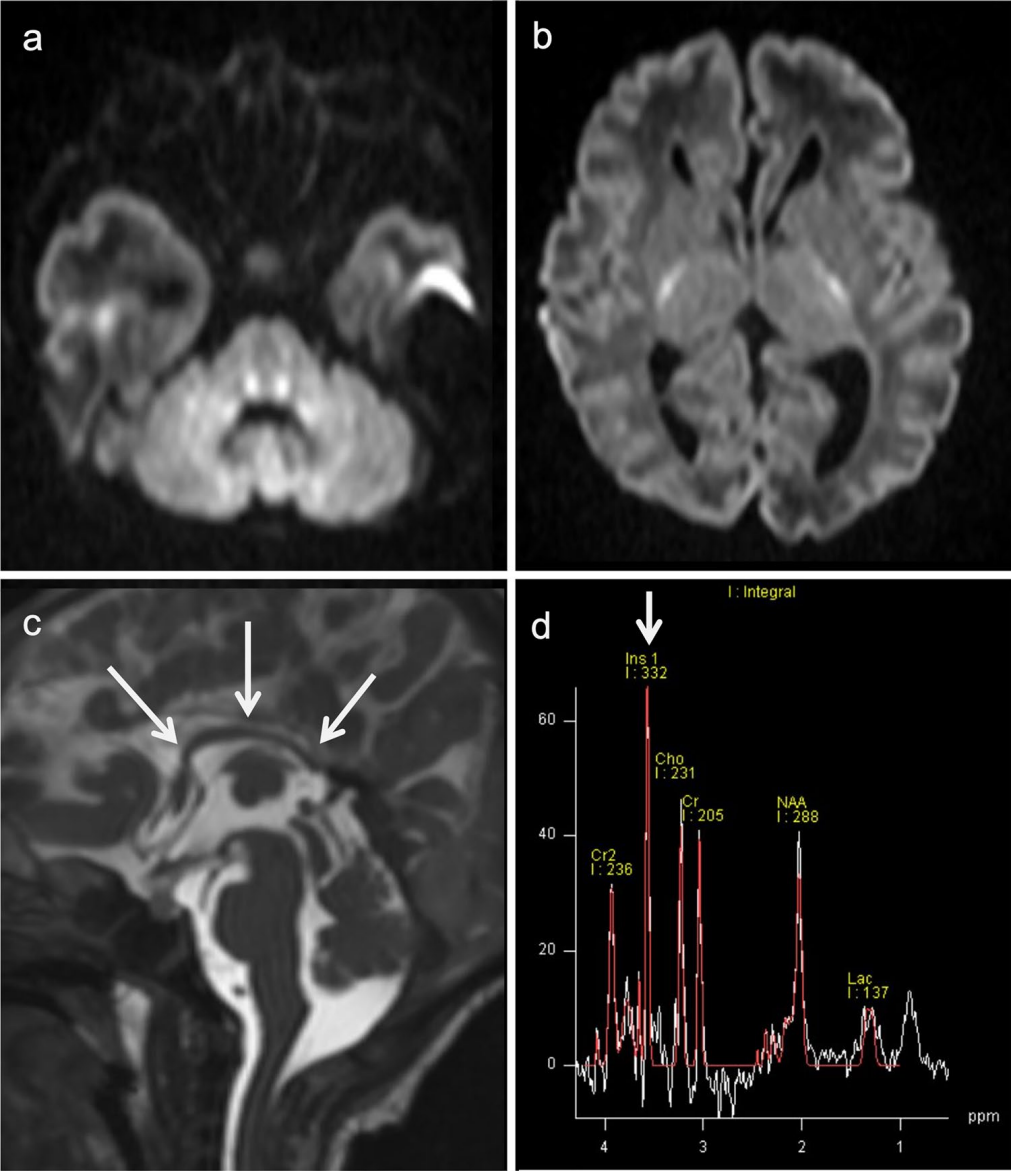
A 6-day-old neonate with nonketotic hyperglycinemia (NKH). **a**, **b** DWI demonstrated symmetrical high signals along the tegmental tracts and posterior limb of the internal capsules. **c** The corpus callosum was small and thin (arrows). **d**. ^1^H-MRS (TE = 30 ms) demonstrated a prominent, high, glycine and mIns peak at 3.55 ppm (arrow), which persisted on long TE MRS (TE = 270) (not shown) (Source: reference 5)

The neuroimaging findings of NKH demonstrate reduced diffusion in myelinated white matter tracts caused by intramyelinic edema and vacuolization (usually involving the internal capsules, brainstem, and cerebellar white matter) though to a lower degree than in MSUD. Additional structural findings usually include hypogenesis of the corpus callosum and hypoplasia of the cerebellar vermis. ^1^H-MRS demonstrates an elevated glycine peak at 3.55 ppm, which is best distinguished from the normal mIns peak with longer TE due by longer T2 decay (Fig. 6) [3, 5, 28].

## Phenylketonuria (PKU)

Phenylketonuria (PKU) is an autosomal recessive disorder caused by mutations in the *PAH* gene at 12q23.2 resulting in elevated phenylalanine. PKU can be detected by NBS in virtually 100% of cases based on the presence of hyperphenylalaninemia [37]. Acute metabolic encephalopathy, a common feature of many aminoacidopathies and organic acidurias, does not occur in PKU. Children with PKU are usually healthy at birth and have normal early development even if untreated. Over time, the classical signs of microcephaly, spasticity, tremor, and athetosis develop, and the patients begin to experience seizures. PKU is frequently accompanied by psychiatric and behavioral problems, such as autistic behavior and attention deficit hyperactivity disorder. Phenylacetic acid may cause lighter pigmentation, eczema, and a musty odor in affected persons. However, children who maintain the target level of plasma phenylalanine via dietary restrictions on phenylalanine intake can be spared severe, cognitive impairment [38].

**Fig. 7:**
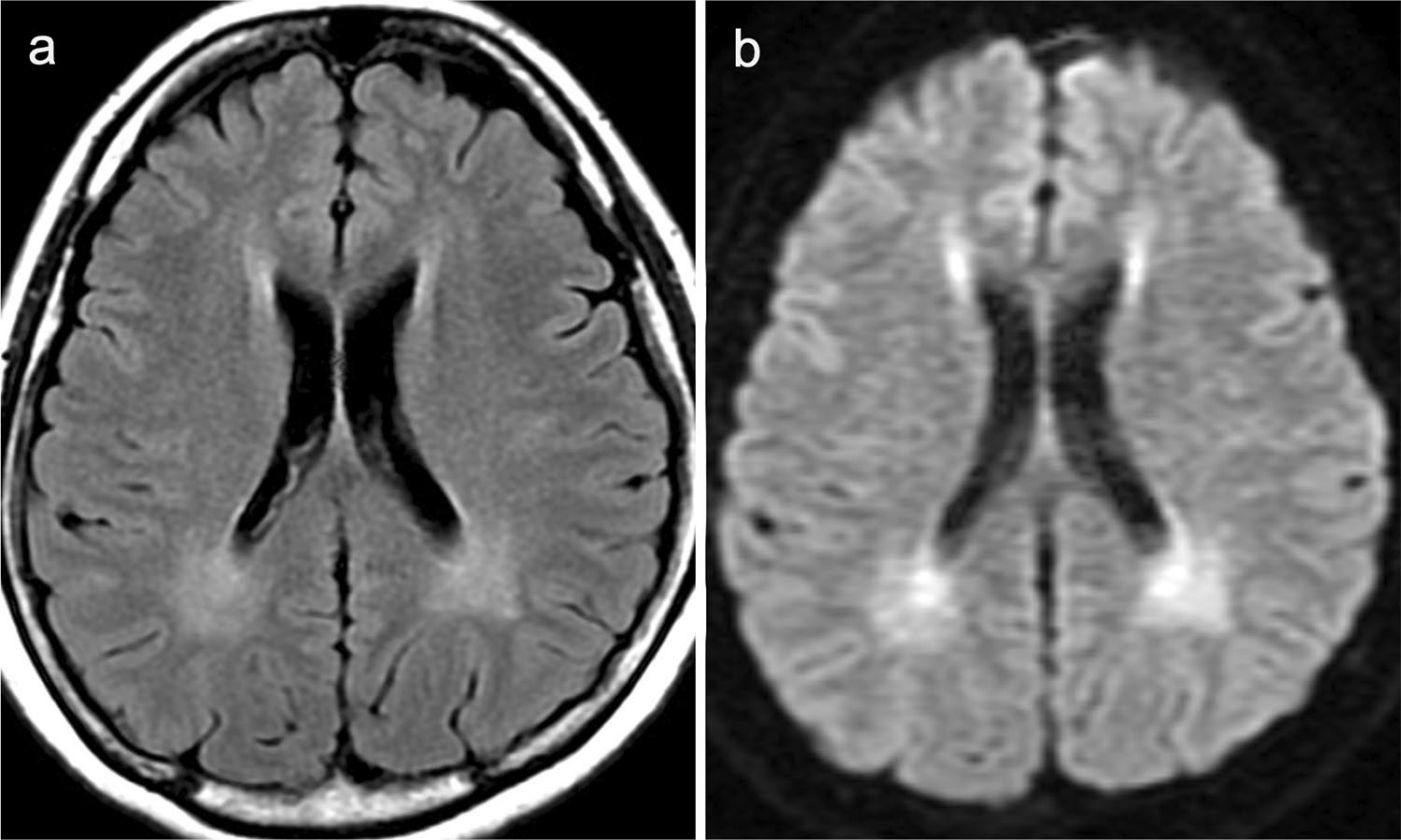
A 32-year-old, female patient with phenylketonuria (PKU). **a** FLAIR image showed increased signal intensity in the periventricular white matter. **b** DWI showed high-signal intensity in the periventricular white matter, indicating restricted diffusion and intramyelinic edema

MRI demonstrates T2/FLAIR hyperintensity in the periventricular white matter which may also affect the subcortical white matter. This high signal intensity probably reflects hypomyelination in untreated patients and intramyelinic edema in patients treated at an early stage of the disease (Fig. 7) [39]. White matter changes seem to be reversible by strictly reducing dietary phenylalanine. ^1^H-MRS with short TE demonstrates a characteristic phenylalanine peak at 7.37 ppm [40, 41] although it is currently not trivial to assess this peak, which is far downfield on most clinical MR scanners.

## Hyperhomocysteinemia (formerly known as homocystinuria)

Homocysteine is the end product of the transmethylation pathway, a multistep, metabolic process by which methyl groups from methionine are transferred to essential acceptor molecules (DNA, neurotransmitters, proteins, phospholipids, polysaccharides). Homocysteine may be either recycled into methionine (via the remethylation pathway) or catabolized into cystathionine, cysteine, and sulfate (via the trans-sulfuration pathway). The three key enzymes involved in the pathogenesis of hyperhomocysteinemia are cystathionine β-synthase, methionine synthase, and 5,10-methylene-tetrahydrofolate reductase. Folate and vitamins B_12_ (cobalamin) and B_6_ (pyridoxine) are cofactors [42].

## Cystathionine β-synthase deficiency (CBSD) (“classic” homocystinuria)

Cystathionine β-synthase deficiency (CBSD), or classic homocystinuria, is an autosomal recessive disorder caused by mutations in the *CBS* gene at 21q22.3. CBSD results in impaired synthesis of cystathionine and an abnormal accumulation of homocysteine, methionine, and other metabolites. Patients present with characteristic clinical abnormalities involving the eye (ectopia lentis and/or severe myopia), skeletal system (excessive height, long limbs, scoliosis, and pectus excavatum), vascular system (thromboembolism), and CNS (developmental delay/intellectual disability, psychiatric problems, seizures, and/or extrapyramidal signs) [43]. Excessive homocysteine generates superoxide and hydrogen peroxide, changes coagulation factor levels, and causes the proliferation of smooth muscle cells in the arterial wall [44]. Thromboembolic events arising from hypercoagulability pose a significant risk of morbidity and mortality and can cause occlusive peripheral venous and arterial diseases, cerebrovascular events, and myocardial infarctions. NBS can identify some individuals with CBSD by detecting hypermethioninemia.

**Fig. 8:**
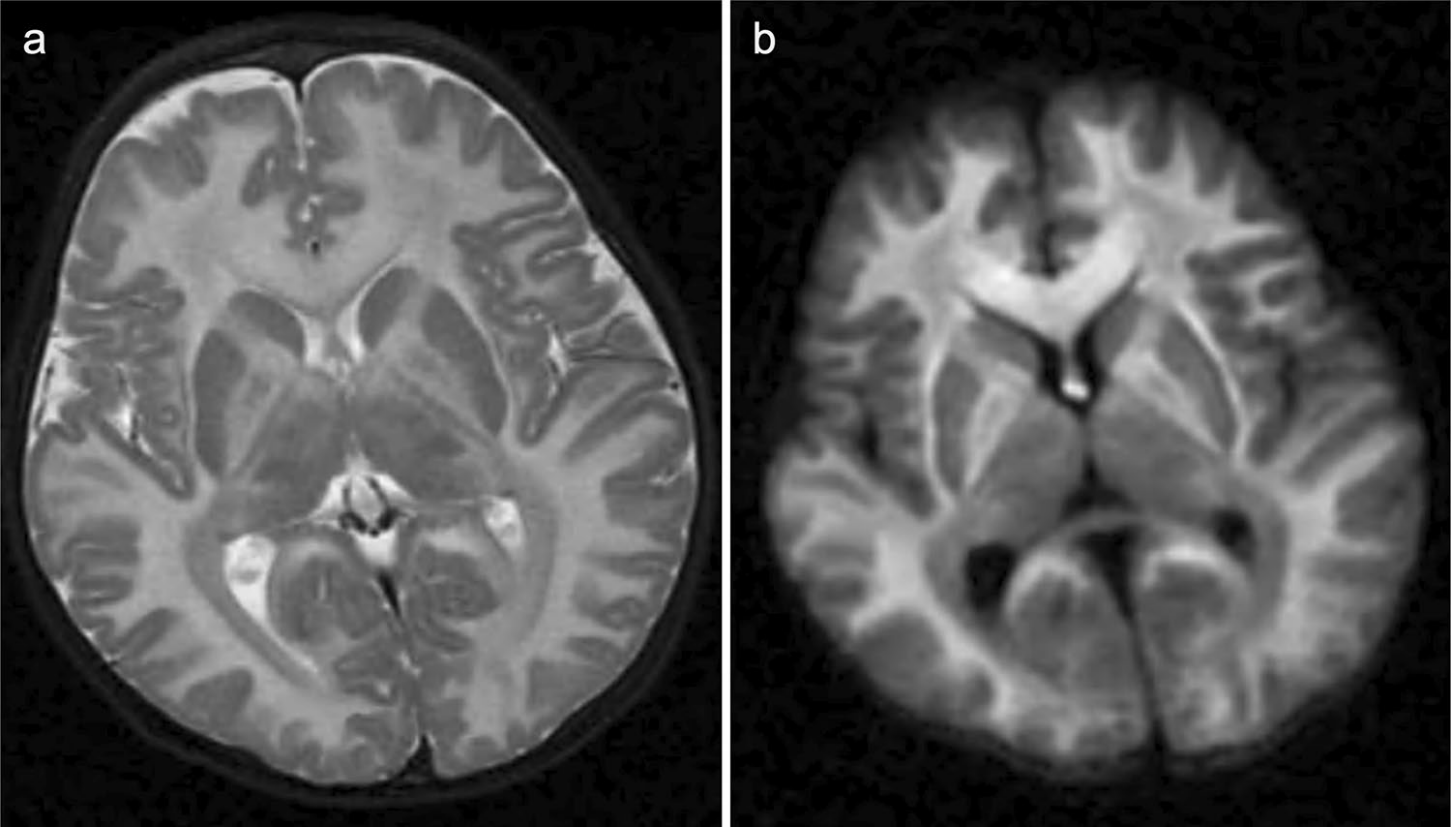
A 2-year-old, male patient with cystathionine β-synthase deficiency (CBSD). **a**, **b** T2-weighted imaging (**a**) and DWI (**b**) demonstrated diffuse T2 hyperintensity and restricted diffusion of the cerebral white matter, corpus callosum, anterior limb of the internal capsules, fornices, and globi pallidi

In most cases, neuroimaging studies demonstrate the presence of multiple, small infarcts incurred at various ages throughout the brain [45]. Dural sinus thrombosis may give rise to secondary venous infarction [46]. Hypermethioninemia-induced demyelination, white matter vacuolization, and spongy degeneration may result in diffuse white matter signal intensity abnormalities and increased intracranial pressure (Fig. 8) [47]. Utilizing MR angiography (MRA) and MR venography (MRV) may aid in detecting occlusive diseases of the large cerebral arteries and intracranial dural sinuses. Furthermore, T2-weighted MRI may reveal intraocular lens dislocation, a symptom highly indicative of CBSD.

## 5,10-Methylene-tetrahydrofolate reductase deficiency (MTHFRD)

5,10-Methylenetetrahydrofolate reductase deficiency (MTHFRD) is a prevalent inborn error of folate metabolism. It is an autosomal recessive disorder caused by mutations in the *MTHFR* gene, which encodes for the eponymous enzyme that plays a vital role in the remethylation pathway, converting homocysteine to methionine. Hence, MTHFRD results in hyperhomocysteinemia and hypomethioninemia. The most common manifestations of MTHFRD are gait abnormalities, seizures, and psychomotor retardation of variable severity, typically presenting in childhood [48]. Infants may also present with hypotonia, progressive lethargy, recurrent apnea, and seizures, and progression to respiratory failure, coma, and death may ensue [49].

Demyelination of the white matter of the brain and spinal cord is the most commonly observed pathology in MTHFRD, which is thought to be associated with S-adenosylmethionine (SAM) deficiency in the CSF [50]. *MTHFR* mutations, including a mild variant (*C677T* polymorphism), are also risk factors of stroke in childhood and cervical arterial dissection [51]; however, this disorder involves a less pronounced vascular pathology than CBSD. It should be noted that patients with a diagnosis of MTHFRD should not be given nitrous oxide as an anesthetic as there is a risk of a nitrous oxide-induced defect in methionine synthase, which, combined with the hereditary defect in *MTHFR,* may lead to neurological deterioration and death [52].

**Fig. 9:**
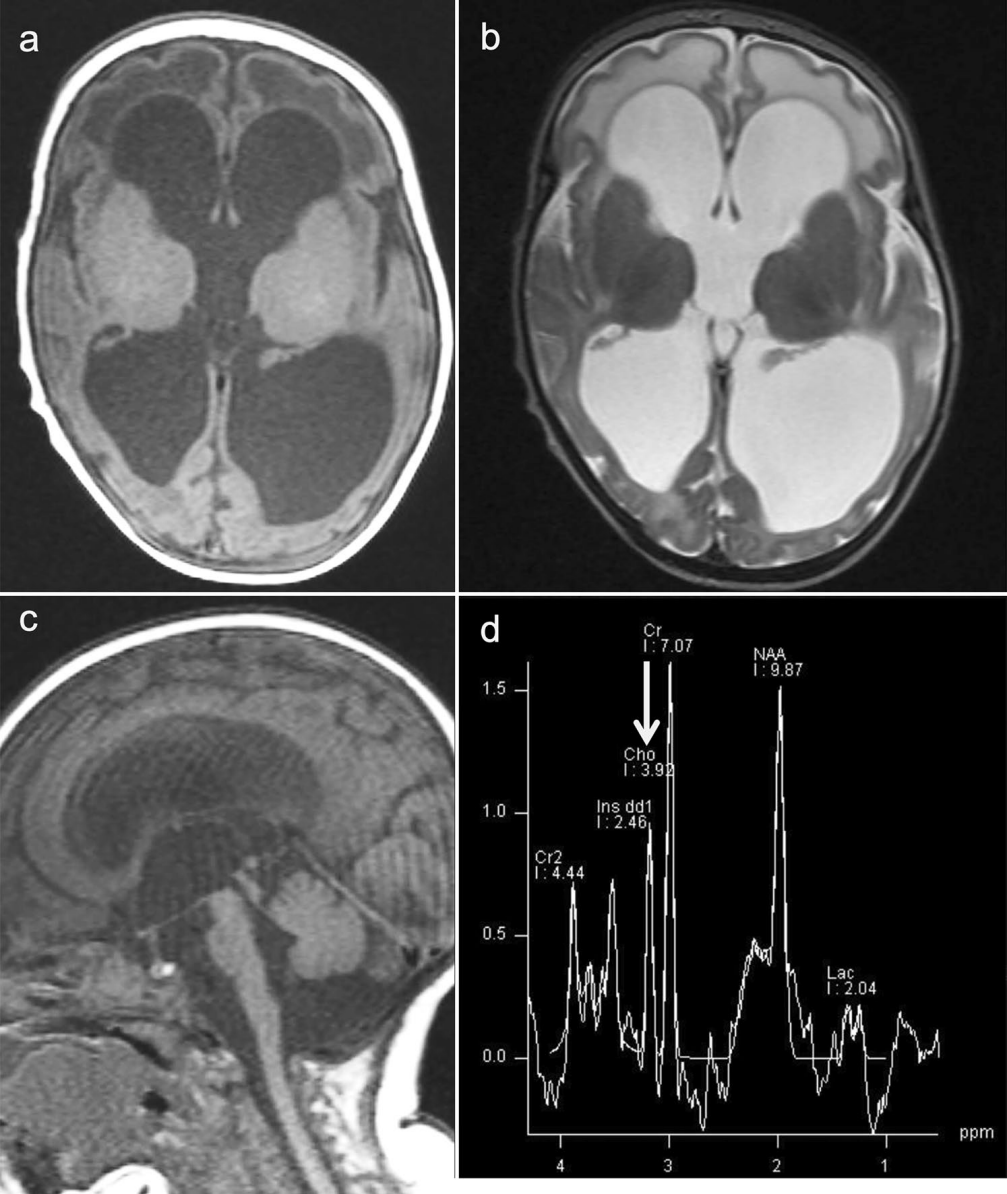
A 2-month-old, female patient with 5,10-methylene-tetrahydrofolate reductase deficiency (MTHFRD). **a** Ax T1-weighted images demonstrated marked dilatation of all ventricles indicating communicating hydrocephalus. **b** T2-weighted images demonstrated hyperintensity in the bilateral frontal periventricular white matter. Delayed myelination can also be seen. **c** Sagittal T1-weighted images demonstrated mild hypoplasia of the pons and cerebellum. **d** ^1^H-MRS (TE = 30) demonstrated a slightly low cho (arrow) and NAA peak (Source: reference 53)

**Fig. 10:**
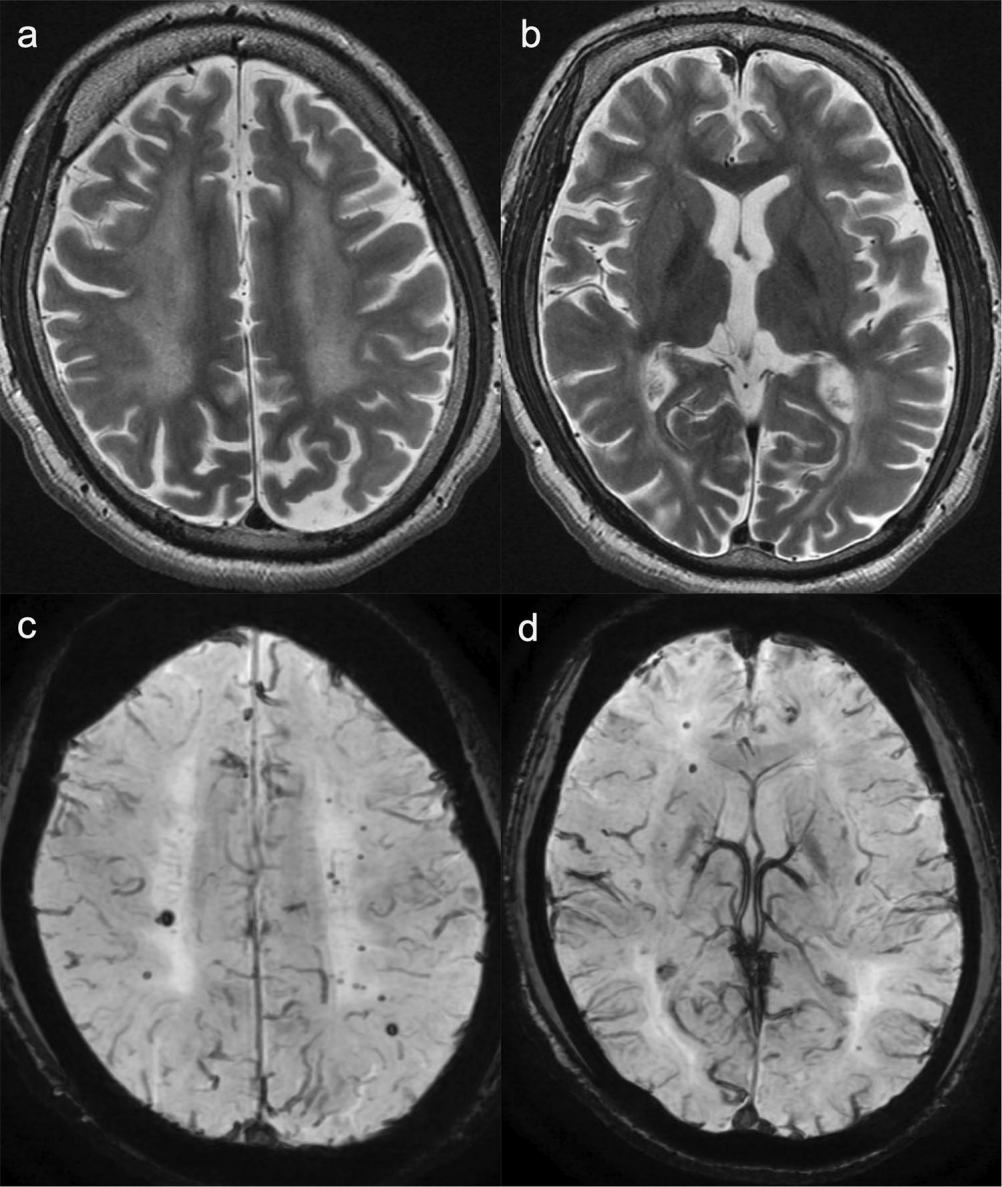
5,10-methylene-tetrahydrofolate reductase deficiency (MTHFRD). An 18-year-old, male patient with epilepsy, intellectual disability, and autistic tendency. The patient had lower limb motor weakness and urinary incontinence for four years. **a**, **b** T2-weighted images demonstrated mild brain atrophy and white matter hyperintensities with posterior predominance. **c**, **d** SWAN demonstrated multiple, cerebral microbleeds in the white matter (Source: reference 55)

In infants with severe MTHFRD, neuroimaging may reveal communicating hydrocephalus, global brain atrophy, and delayed myelination (Fig. 9) [49, 53]. In children and adults with MTHFRD, neuroimaging demonstrates white matter abnormalities ranging from small foci to more diffuse areas of high signal intensity indicating demyelination (Fig. 10) [54, 55]. In addition, areas of vascular infarction, venous thrombosis [56], and/or cerebral microbleeds may be seen. Less commonly, demyelination of the dorsal and lateral columns of the spinal cord may be present [57]. A slightly decreased cho and NAA peak level on ^1^H-MRS has been reported [58, 59]. Decreased cho is probably secondary to the depletion of labile methyl groups produced by the transmethylation pathway.

## Organic acidopathies

### Propionic acidemia (PA)

Propionic acidemia (PA) is an autosomal recessive disorder caused by deficiency of propionyl-CoA carboxylase (PCC), a mitochondrial, multimeric enzyme which catalyzes the conversion of propionyl-CoA to D-methylmalonyl-CoA. Deficient PCC activity results in the accumulation of propionic acid and propionyl-CoA-related metabolites. Infants at risk of PA can be detected via NBS although symptoms of the condition may appear before NBS results are available [60].

The onset of PA can occur at any time from birth to late in life. The most common form, neonatal-onset PA, starts with poor feeding and decreased arousal in the first few days of life. Without prompt diagnosis and treatment, this can lead to progressive encephalopathy and potentially fatal symptoms, such as lethargy, seizures, and coma. PA also often causes metabolic acidosis, lactic acidosis, ketonuria, hypoglycemia, hyperammonemia, and cytopenia. Late-onset PA can be asymptomatic until a metabolic crisis occurs under stress (e.g., illness, surgery, fasting) or can cause symptoms, such as vomiting, protein intolerance, failure to thrive, hypotonia, developmental delays, movement disorders, and cardiomyopathy [61].

**Fig. 11:**
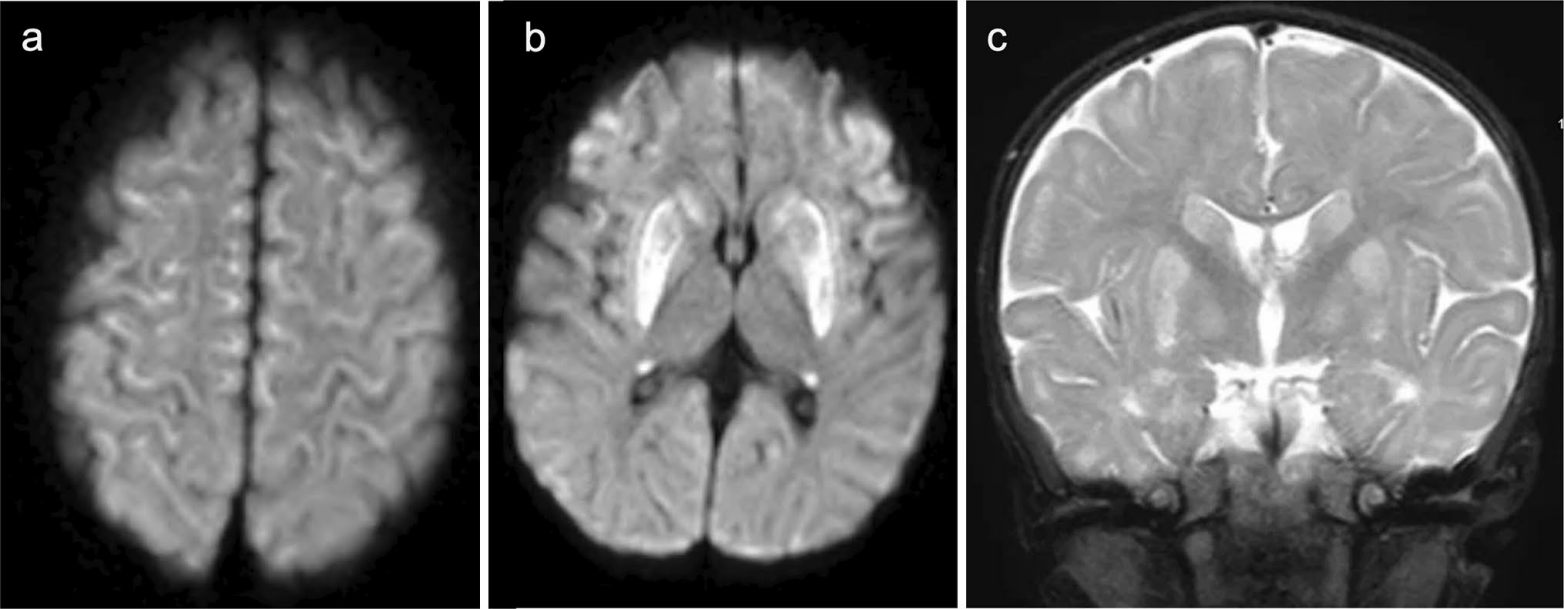
An 8-month-old, female patient during acute decompensation with propionic acidemia (PA). **a**, **b** DWI showed restricted diffusion in the bilateral cerebral cortices, putamina, and caudate nuclei. **c** Coronal T2-weighted imaging demonstrated mild swelling and increased signal of the bilateral cerebral subcortices, putamina, caudate nuclei, and globi pallidi

**Fig. 12:**
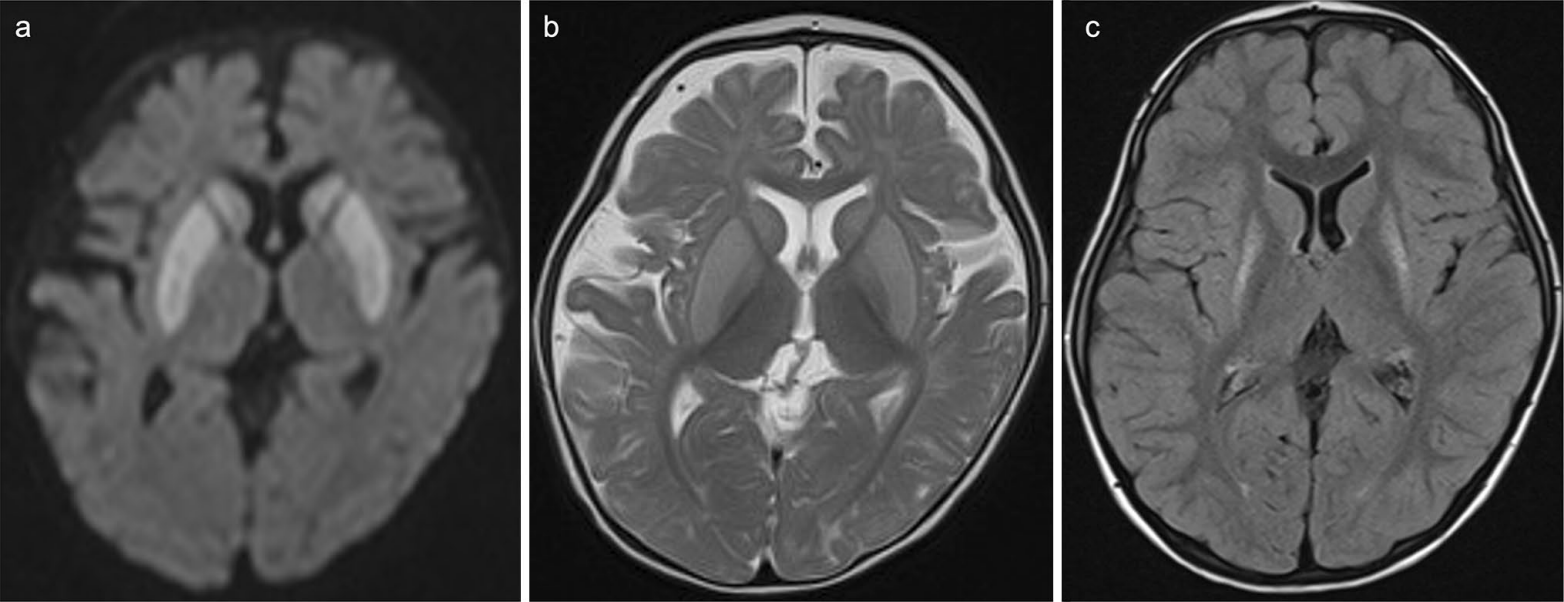
An 8-month-old, female patient during acute decompensation with propionic acidemia (PA). **a**,**b** DWI (**a**) and T2-weighted imaging (**b**) displayed restricted diffusion and hyperintensities in the bilateral putamina and caudate nuclei. **c** Four years later, FLAIR imaging revealed atrophy and increased signal intensity in the bilateral putamina

In affected neonates, acute neuroimaging findings may reveal diffuse brain swelling with restricted diffusion in the internal capsule and the adjacent globi pallidi, hippocampi, and mesencephalon [3], indicating white matter spongiosis [62]. The basal ganglia may be normal in PA during the neonatal period. In older children with acute decompensation, the putamina and caudate nuclei of the basal ganglia and less frequently the substantia nigras and dentate nuclei show areas of hyperintense signaling as well as reduced diffusion on T2-weighted and FLAIR MR imaging (Figs. 11, 12) [63]. The cerebral and cerebellar cortices and the subcortical white matter may also display abnormal signal intensity and mild swelling (Fig. 11). Intracranial hemorrhages may occur [64]. During the chronic stage, delayed myelination and later-onset cortical and white matter atrophy with signal intensity changes in the basal ganglia are usually seen (Fig. 12). During the acute decompensation stage, ^1^H-MRS may demonstrate decreased NAA, decreased Glx, and increased Lac during encephalopathic episodes [65].

### Methylmalonic acidemia (MMA)

Methylmalonic acidemia (MMA) is an autosomal recessive disorder caused by deficiency of the enzyme methylmalonyl-CoA mutase, a defect in the transport or synthesis of its cofactor, adenosyl-cobalamin (vitamin B_12_) (*cblA*, *cblB*, or *cblD*-MMA) or deficiency of the enzyme methylmalonyl-CoA epimerase.

The onset of MMA symptoms can range from the neonatal period to adulthood. MMA is characterized by periods of relative health and intermittent metabolic decompensation, which are usually associated with intercurrent infections and stress. The most severe form of MMA, neonatal-onset MMA, may present with symptoms such as lethargy, vomiting, hypotonia, hypothermia, respiratory distress, severe ketoacidosis, hyperammonemia, neutropenia, and thrombocytopenia, which can lead to death within the first four weeks of life. Secondary complications of MMA may include intellectual impairment, tubulointerstitial nephritis with progressive renal failure, and movement disorders, such as choreoathetosis, dystonia, and para/quadriparesis, pancreatitis, growth failure, functional immune impairment, and optic nerve atrophy [61, 66]. MMA can be diagnosed prior to an episode of acute decompensation via NBS [66].

**Fig. 13:**
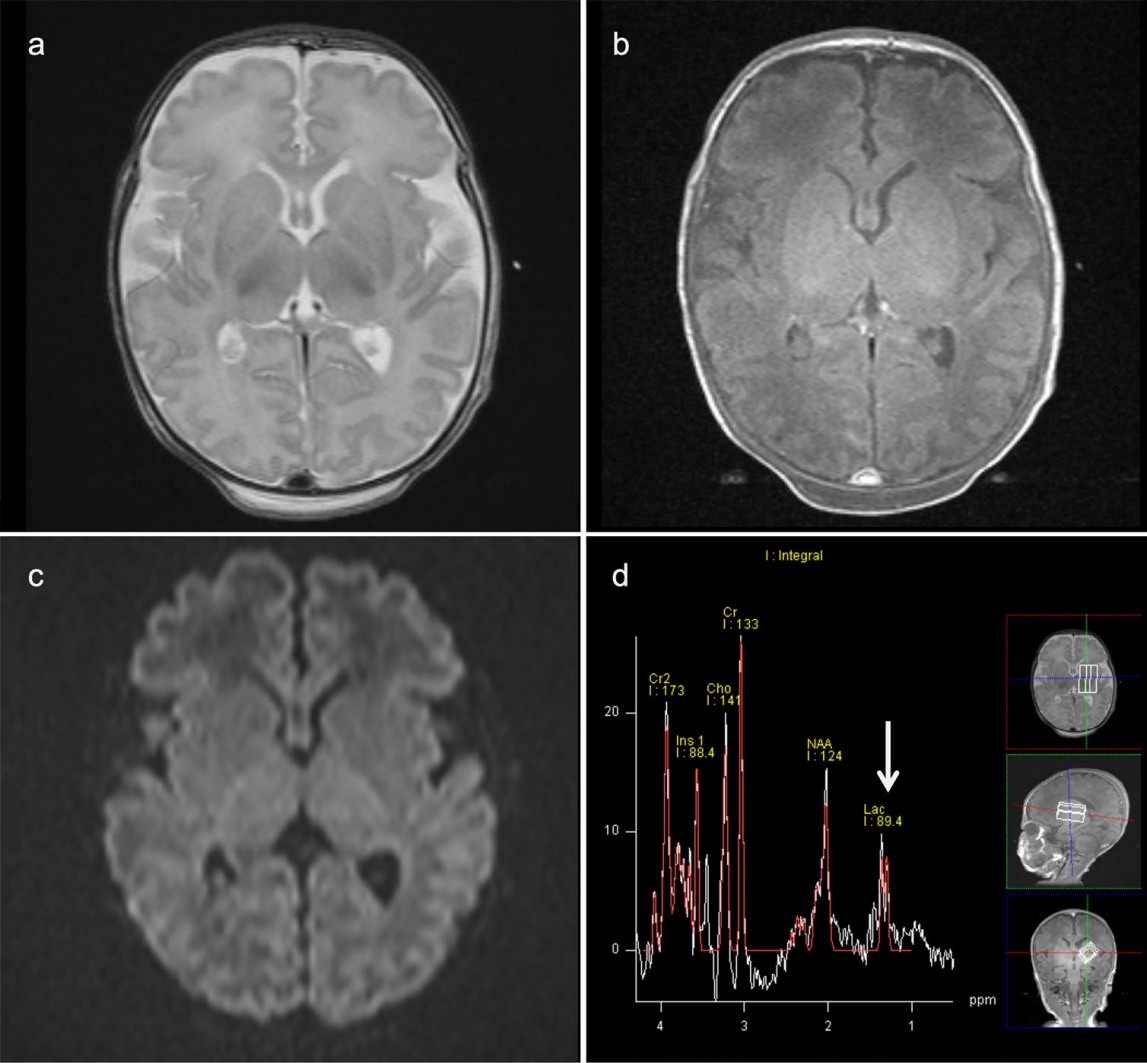
An encephalopathic neonate with methylmalonic acidemia (MMA). **a**, **b** T2- (**a**) and T1-weighted images (**b**) indicated diffuse T1 and T2 prolongations in the cerebral white matter. **c** No restricted diffusion was visible, but the white matter showed a relatively a low DWI signal. **d** ^1^H-MRS (TE = 30) demonstrated elevated Lac (arrow) and decreased NAA (Source: reference 5)

**Fig. 14:**
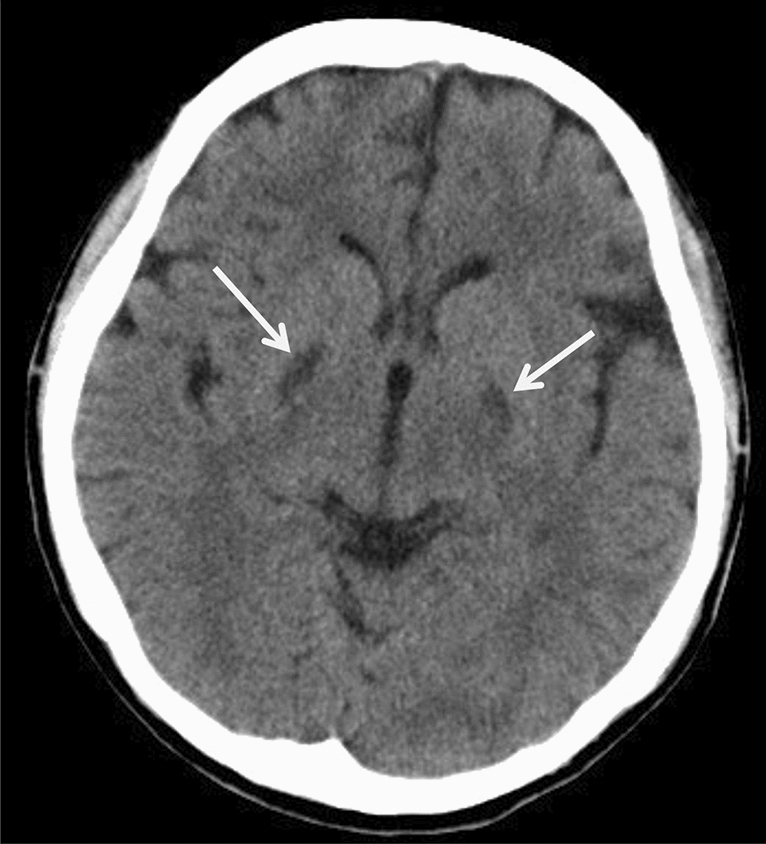
A 25-year-old, female patient with methylmalonic acidemia (MMA) and chronic renal failure. CT showed abnormally low density in the globi pallidi (arrows)

Neuroimaging in neonatal MMA may demonstrate diffuse, T2-hyperintense brain swelling with or without a matching, reduced diffusivity (Fig. 13) [3, 5, 67] while later imaging reveals brain atrophy, ventricular dilatation, thinning of the corpus callosum, periventricular and subcortical white matter abnormalities, myelination delay, calcification of the basal ganglia, and focal necrosis of the globi pallidi (Fig. 14) [68]. In addition, intracranial hemorrhages have also been reported [64]. ^1^H-MRS can detect reductions in mIns and NAA concentrations. Elevation of Glx and Lac concentrations secondary to hyperammonemia, ketoacidosis, and mitochondrial dysfunction have been reported in patients during a metabolic crisis [5, 69].

### Glutaric aciduria type 1 (GA1)

Glutaric aciduria type 1 (GA1) is an autosomal recessive disorder caused by deficiency of the enzyme, glutaryl-CoA dehydrogenase (GCDH), which leads to the accumulation of glutarate, glutarylcarnitine, 3-hydroxyglutarate, and glutaconate in various tissues, blood, CSF, urine, and multiple organs [70]. Elevated levels of glutarate and 3-hydroxyglutarate may cause an imbalance in glutamatergic and γ-aminobutyric acid-mediated neurotransmission, and 3-hydroxyglutarate can cause excitotoxic cell damage through the activation of N-methyl-d-aspartate receptors, resulting in striatal lesions [71].

Symptoms typically include acute encephalopathy in infancy with dystonia, dyskinesia, and long-term motor and language impairment. Intelligence may be relatively preserved. GA1 can be detected before symptoms via NBS by measuring glutarylcarnitine in dried blood spots [72]. A therapeutic regimen in the pre-encephalopathic state is important for normal development or mitigating neurological sequelae.

**Fig. 15:**
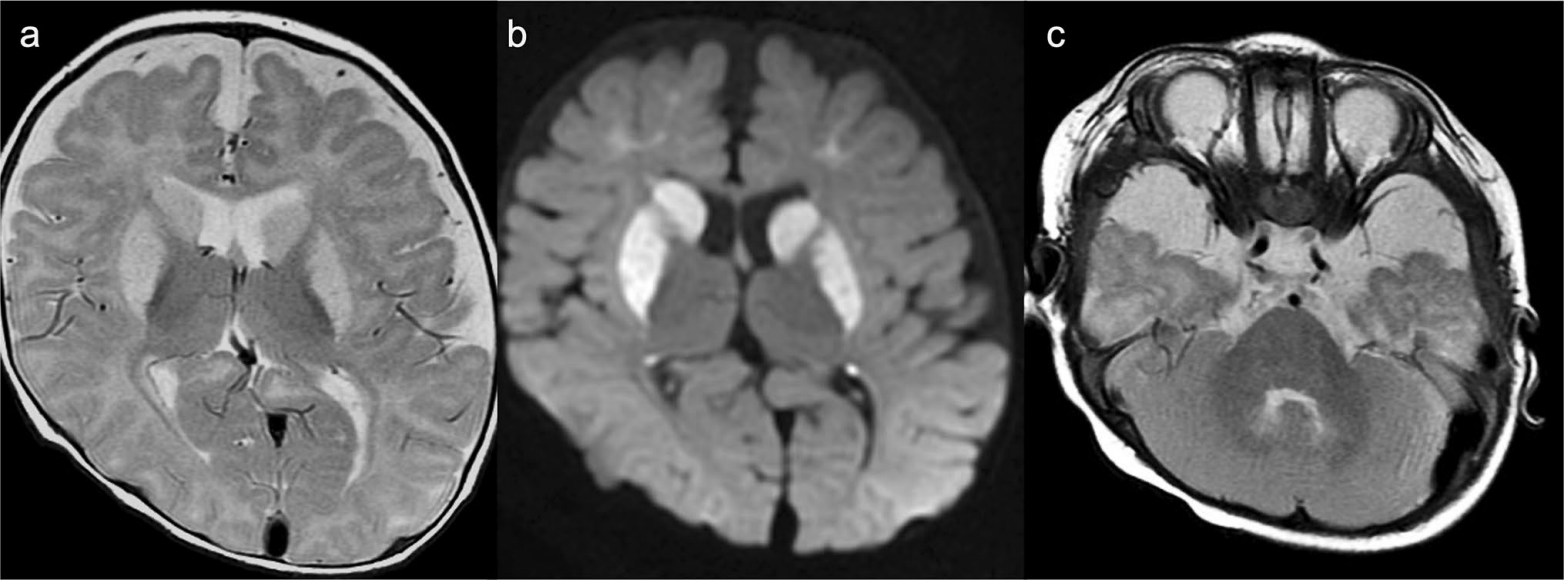
Glutaric aciduria type 1 (GA1). A 6-month-old, male patient with eye deviation, cyanosis, and myoclonus. **a**, **b** T2-weighted images (**a**) and DWI (**b**) demonstrated increased high signal and restricted diffusion of bilateral caudate nuclei and putamina. **c** T2-weighted images demonstrated enlargement of the Sylvian fissures caused by hypoplasia of the temporal opercula

The characteristic findings of GA1 on neonatal neuroimaging are enlarged frontotemporal CSF spaces, wide Sylvian fissures, and a large cavum septi pellucidi [73]. Symptomatic children have symmetric injury to the basal ganglia (mostly the putamen and caudate), whereas globus pallidum involvement is less common (Fig. 15) [73, 74]. The basal ganglia show swelling and restricted diffusion during the acute phase and become atrophic with disease progression. Putaminal changes on MRI are reliable predictors of long-term movement disorders [74]. In addition, abnormalities of the upper brainstem showing increased signal intensity in the substantia nigras, central tegmental tracts, tegmentum, dentate nuclei, and white matter on T2-weighted MRI may occur [28].

Note that chronic subdural effusions and hematomas mimicking abusive head trauma can be seen in symptomatic and asymptomatic GA1 patients [73]. The hematomas are presumably related to the rupture of bridging veins with minimal trauma in the presence of cerebral atrophy and enlargement the subarachnoid spaces. Retinal hemorrhage also has been reported [75]. ^1^H-MRS may reveal a mild increase in the Lac level during the acute phase whereas the NAA level is typically depressed during the subacute and chronic phases [76].

### L-2-Hydroxyglutaric aciduria (L2HGA)

2-Hydroxyglutaric aciduria (2HGA) is a rare, neurometabolic disorder which includes D2HGA, L2HGA, and combined D-L2HGAs. L2HGA is an autosomal recessive disorder caused by mutations in *L2HGDH*, a gene encoding a mitochondrial enzyme, which cause the accumulation of l-2-hydroxyglutaric acid in urine, CSF, and plasma [77]. Affected individuals have neurological manifestations, including mild to moderate psychomotor retardation, hypotonia, cerebellar ataxia, variable macrocephaly, and epilepsy within the first year of life [78], sometimes followed by dystonia and pyramidal signs. L2HGA progresses slowly and rarely leads to an early loss of gross motor skills, such as unaided sitting or walking. Most patients attain adulthood. Increased occurrence of brain tumors has been reported in association with this disease [79].

**Fig. 16:**
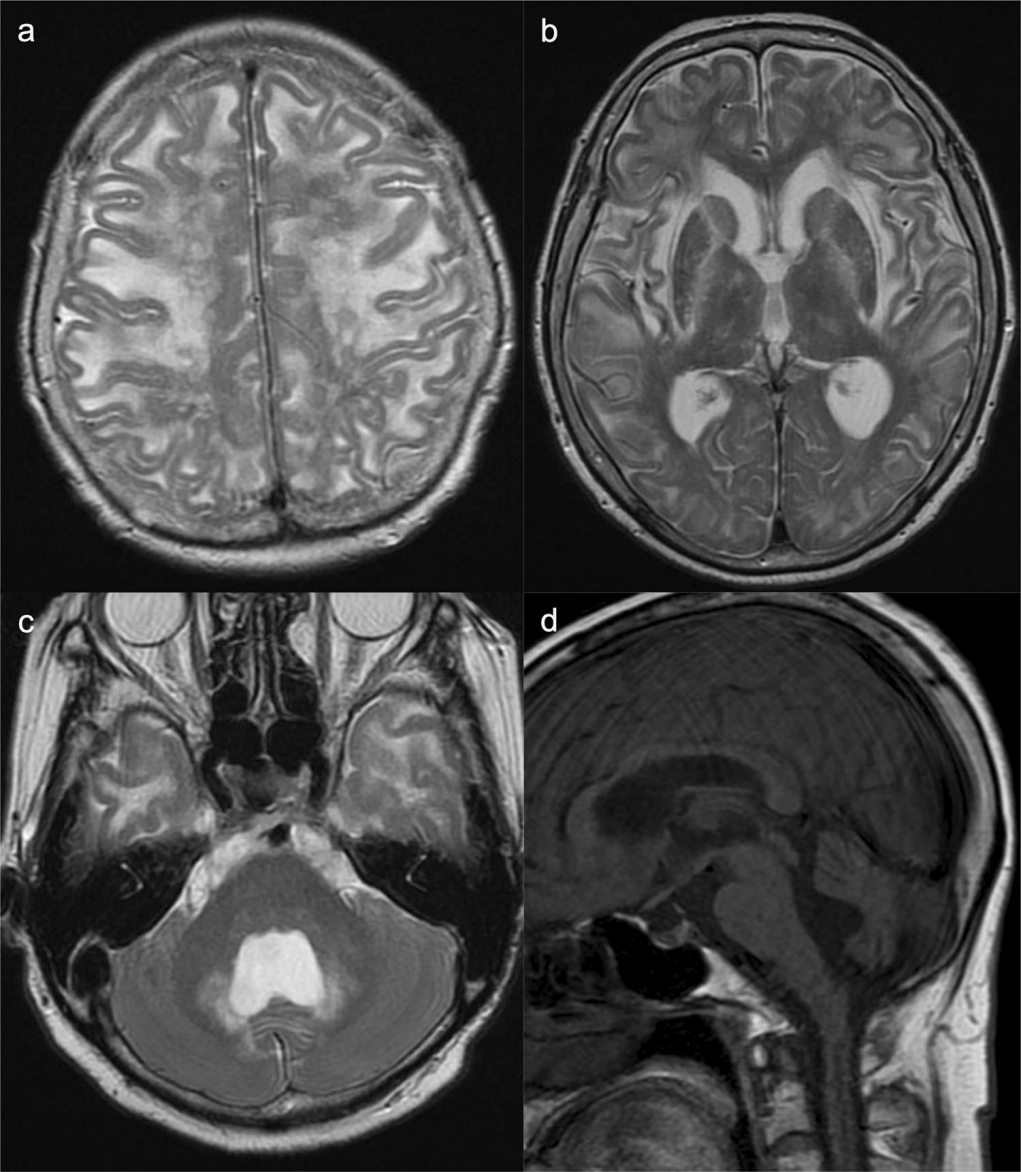
A 69-year-old, female patient with L-2-hydroxyglutaric aciduria (L2HGA). Mental retardation was present since early childhood. She had a resting tremor of the left upper extremity, gait disturbance, and involuntary movements. **a**–**c** T2-weighted images demonstrated increased signal intensity in the subcortical white matter, anterior limb of the internal capsules, globi pallidi, external capsules, and dentate nuclei. **d** Sagittal T1-weighted image showed cerebellar atrophy

MRI findings of L2HGA are pathognomonic for the disease. T1 and T2 prolongation of cerebral white matter exhibits a characteristic, centripetal, slightly anteroposterior gradient. The subcortical white matter is most severely affected [78, 80] whereas the periventricular white matter, corticospinal tracts, and corpus callosum are spared. T1 and T2 prolongation is also seen in the globi pallidi and cerebellar nuclei. The extreme and external capsules, as well as the anterior limb and genu of the internal capsules, are abnormal (Fig. 16). Pathological analysis of affected brains reveals spongiform degeneration of the cerebral and cerebellar white matter with intense gliosis and vacuolation of neuropiles [81]. However, unlike in Canavan disease, the brainstem and thalami are usually spared. Cerebellar atrophy may occur [78, 80]. ^1^H-MRS may reveal mildly decreased NAA and increased mIns [82].

## Conclusion

In the present review, we described neuroimaging findings of IEMs, focusing on UCDs, aminoacidopathies, and organic acidopathies. Radiologists, neuroradiologists, and pediatric neurologists should be familiar with the neuroimaging findings of IEMs that diagnose the diseases correctly and administer early treatment.
